# Better Gingival Status in Patients with Comorbidity of Type 1 Diabetes and Thyroiditis in Comparison with Patients with Type 1 Diabetes and No Thyroid Disease—A Preliminary Study

**DOI:** 10.3390/ijerph20043008

**Published:** 2023-02-09

**Authors:** Anna Duda-Sobczak, Dorota Zozulinska-Ziolkiewicz, Marzena Wyganowska

**Affiliations:** 1 Department of Internal Medicine and Diabetology, Poznan University of Medical Sciences, Mickiewicza 2, 60-830 Poznan, Poland; 2 Chair and Department of Dental Surgery, Periodontology and Oral Mucosa Diseases, Poznan University of Medical Sciences, Bukowska 70, 60-812 Poznan, Poland

**Keywords:** gingivitis, thyroiditis, type 1 diabetes, microvascular complications

## Abstract

Periodontal disease has been postulated as one of the chronic complications of diabetes. The prevalence of autoimmune thyroiditis in type 1 diabetes (T1D) is higher. The aim of the study was to determine the association between the presence of thyroiditis and gingival status in adults with T1D. A total of 264 patients, 119 men aged 18–45, diagnosed with T1D were included. For further analysis, the study group was divided into two subgroups, with or without autoimmune thyroiditis. Gingival status was assessed with the use of gingival indices. Patients diagnosed with T1D and thyroiditis presented lower plaque accumulation (*p* = 0.01) and lower-grade gingivitis (*p* = 0.02). Approximal Plaque Index (API) in all study groups correlated positively with age (Rs = 0.24; *p* = 0.0001), body mass index (BMI) (Rs = 0.22; *p* = 0.0008), hemoglobin A1c (HbA1c) (Rs = 0.18; *p* = 0.006), high-sensitivity C-Reactive Protein (hsCRP) (Rs = 0.17; *p* = 0.009), total cholesterol (T-Chol) (Rs = 0.17; *p* = 0.01) and negatively with thyroid-stimulating hormone (TSH) (Rs = −0.2; *p* = 0.02). Stepwise multivariate linear regression analysis indicated TSH, BMI and gender as independent predictors of dental plaque accumulation in patients with T1D. Autoimmune thyroiditis was associated with a lower accumulation of dental plaque and better gingival status in patients with T1D.

## 1. Introduction

Prolonged exposure to hyperglycaemia leads to the development of neurovascular complications such as retinopathy, chronic kidney disease, and neuropathy. Subjects diagnosed with diabetes are also at higher risk of developing periodontal disease. Chronic hyperglycemia and oxidative stress lead to an imbalance between the opportunistic flora of the dental biofilm and the host immune response and to the development of chronic inflammation and destruction of the teeth-supporting tissues [1]. Periodontal disease is one of the manifestations of chronic inflammation in diabetes and may be classified as a chronic complication of the disease. It is suggested that gingival inflammation in young adults with T1D is an overt response to the dental biofilm [2]. A meta-analysis of interventional studies showed that control of inflammation in gingiva would improve glycemic control in patients with type 2 diabetes, but the results are not obvious in patients with type 1 diabetes [3]. Pathogenic ground for the development of gingivitis or periodontitis in T1D has not been fully explained since the periodontal disease does not appear in every patient with metabolically uncontrolled diabetes. It can be assumed that other factors or coexisting diseases may alter the risk. The prevalence of thyroid dysfunction in T1D, most frequently autoimmune thyroiditis, is higher than in the general population [4,5,6]. The role of thyroid hormones in alveolar bone metabolism during periodontitis is also described [7]. However, the significance of the coexisting autoimmune thyroiditis and T1D in the assessment of the risk of gingivitis has not been evaluated. The aim of the present study was to determine the association between the presence of thyroiditis and gingival status in adults with T1D.

## 2. Materials and Methods

This study was performed in the Department of Internal Medicine and Diabetology, Poznan. The inclusion criteria were: subjects with T1D aged 18–45, signed informed consent. Exclusion criteria: diagnosed advanced chronic complications of diabetes causing disability (limb amputation, blindness, end-stage renal disease), acute complications of diabetes (symptomatic hypoglycemia, diabetic ketoacidosis), medical history of maxillofacial surgery. We included 264 patients, 119 men aged 18–45, diagnosed with T1D, who had remained under medical care at the Department. The ethical committee of the Poznan University of Medical Sciences approved the study protocol, and written informed consent was obtained from all subjects prior to their participation in the study. The project was conducted according to the rules of the Helsinki Declaration.

### 2.1. Evaluation of Type 1 Diabetes

T1D was diagnosed according to the presence of classical symptoms at the onset, blood glucose concentration above 11.1 mmol/l and the presence of at least one of the assessed autoantibodies: islet cells (ICA), glutamic acid decarboxylase (anti-GAD), insulinoma-associated tyrosine phosphatase (IA-2A) autoantibodies.

All patients underwent a complete physical examination with anthropometric measurements.

We assessed the metabolic control of diabetes and the presence of diabetic retinopathy and neuropathy. Diabetic retinopathy was diagnosed using direct ophthalmoscopy through dilated pupils. Neuropathy assessment was performed using pressure sensation (10-g monofilament perception), vibration perception (128-Hz tuning fork), and ankle reflex tests. Diabetic neuropathy was diagnosed in patients with two or more of the following four components: the presence of typical symptoms of neuropathy, the absence of ankle tendon reflexes, and/or abnormal scores for pressure and/or vibration perception.

Blood samples were obtained after an overnight fast. Serum lipids and creatinine were measured with standard techniques. Hemoglobin A1c (HbA1c) was measured using the high-performance liquid chromatography method aligned to the Diabetes Control and Complications Trial DCCT standard.

### 2.2. Evaluation of Thyroid Function

Serum thyroid-stimulating hormone (thyrotropin, TSH) level was assessed using electrochemiluminescence ECLIA Elecsys analyzers. The normal range for TSH was 0.27–4.2 mU/L. The evaluation of TSH was performed in patients in the stable metabolic state of glycaemia between 70 and 180 mg/dl and with no ketonuria.

Medical records were checked in all patients previously diagnosed with thyroid disease. According to the type of thyroid dysfunction, only patients with documented positive anti-thyroperoxidase autoantibodies (ATPO) and/or antithyroglobulin antibodies (ATg) were included in the study. We collected data on the supplementary dose of thyroxin.

In the T1D group without previously diagnosed thyroiditis, ATPO and ATg were determined by the ARCHITECT ATPO and ATg assays to exclude the cases of undiagnosed thyroiditis. Patients with negative ATPO and ATg results were included in T1D without thyroiditis group.

### 2.3. Evaluation of Periodontal Status

Clinical examination of periodontal tissue was provided by the same periodontologist to avoid differences in measurement. WHO probe was used. The severity of gingivitis was described by Gingival Index, GI [8]. In the GI scale code, 0 means no bleeding and inflammation; code 1—a slight change in color and texture and mild oedema, mild inflammation; code 2—redness, hypertrophy, oedema and glazing, bleeding on probing, moderate inflammation, and code 3—marked redness, hypertrophy, oedema, ulceration, spontaneous bleeding, severe inflammation. The activity of inflammation was described by a modified Sulcus Bleeding Index, SBI [9]. In the SBI scale, severe gingivitis is recognized when SBI is between 100–50%, moderate gingivitis when SBI is between 50–20%, mild gingivitis when the score is 20–10% and no inflammation when SBI is below 10%. The hygiene level was described by Approximal Plaque Index, API [10]. API 100–70% indicated bad oral hygiene, 70–40% average hygiene, 39–25% almost good, and below 25% good oral hygiene.

### 2.4. Statistical Analysis

We divided the study group into two subgroups according to diagnosed thyroiditis. Results are presented as median (interquartile range 25–75%, IQR) for continuous variables or number (proportion) of patients for categorical variables. Correlation analysis was used to study relationships between various variables. Mann-Whitney U test was used to analyze the differences between subgroups according to the presence of thyroiditis. The chi-square test was used to compare frequencies.

There were no differences in bleeding (SBI) between subgroups; still, the significant differences in plaque accumulation (API), we chose to use API in the further analysis. Stepwise multiple regression was used to assess the effect of thyroid function (expressed as TSH level) on dental plaque accumulation. Based on clinical judgment, we included the following confounders in the stepwise models: age, gender, cigarette smoking, BMI, HbA1c, and T-Chol.

A *p*-value of less than 0.05 was considered statistically significant. Statistical analysis was performed using STATISTICA 10 (StatSoft, Tulsa, Oklahoma, USA).

## 3. Results

The clinical characteristics of the study group and analyzed subgroups according to diagnosed thyroiditis are presented in Table 1.

No age differences were detected between subgroups with and without thyroiditis. In the subgroup with T1D and thyroiditis, there was a higher prevalence of women (*p* = 0.00001). Patients diagnosed with T1D and thyroiditis had better metabolic control of diabetes (HbA1c 8.1 (7.4–9.4)% vs. 7.7 (7.1–9.05)%; *p* = 0.049) and less frequently smoked cigarettes (*p* = 0.0001). According to the gingival status, we found statistically significant lower plaque accumulation and lower-grade gingivitis in the thyroiditis subgroup Table 2.

We found no gender differences in dental plaque accumulation (API) within each subgroup and no differences when comparing men and women from each subgroup separately. There were no differences in sulcus gingival bleeding (SBI) between subgroups, and SBI did not correlate with API either in the study group (Rs = 0.03; *p* = 0.6) or in the subgroups of thyroiditis (Rs = 0.08; *p* = 0.5) and non-thyroiditis (Rs = 0.04; *p* = 0.5). Dental plaque accumulation (API) in the study group correlated positively with age, BMI, HbA1c, hsCRP, and T-Chol and negatively with TSH Table 3.

Stepwise multivariate linear regression analysis indicated TSH, BMI and gender as independent predictors of dental plaque accumulation in patients with T1D Table 4.

## 4. Discussion

Gingivitis and periodontitis are a continuum of the same inflammatory disease [11], and while not all patients with gingivitis will progress to periodontitis, both are modified by diabetes. Periodontitis is also described in children with type 1 diabetes [12,13] and as a manifestation of diabetes in adolescence [14]; however, in our group aged between 18–45, we detected gingivitis only. Moreover, we did not observe a correlation between plaque accumulation and bleeding on probing in both subgroups, but patients with T1D and thyroiditis had lower dental plaque accumulation and better clinical status of gingiva compared to the non-thyroiditis group. During the clinical examination of study participants, we observed bleeding on probing even when no dental plaque was present, which can suggest an earlier reaction of gingival tissue to plaque irritation in patients with diabetes. This observation is consistent with other reports [2,15]. Plaque accumulation is generally associated with tooth-brushing habits. In our previous study, we showed that the bleeding score was independent of oral hygiene in patients with T1D and was associated with long-term metabolic control of diabetes (the assessment of the tissue accumulation of advanced glycation end products (AGEs) by measuring skin autofluorescence, AF) [16]. According to the published data, the TSH level correlates with the vessel’s condition [17]. This might explain no differences in SBI in both examined groups and the difference in clinical signs of inflammation in both groups defined by the GI index, which includes the observation of bleeding as well as clinical signs of inflammation. Patients with T1D and thyroiditis had better GI indices. In case of no differences in bleeding, it could suggest the significance of hygiene status in those groups. Therefore we finally decided to use API in the multivariate analysis. It is well known that chronic hyperglycaemia in diabetes creates a microvascular environment in the gingiva that is, in many aspects, comparable to acute and chronic inflammation, with increased vascular permeability, increased leukocyte adhesion molecule expression, and enhanced leukocyte rolling [18]. In our previous study, patients with poorly controlled T1D had higher dental plaque accumulation compared to patients with good metabolic control of diabetes [19]. In the present study, HbA1c was significantly lower in the thyroiditis group in comparison with the non-thyroiditis group, and HbA1c correlated positively with API. Better metabolic control of diabetes may partly explain the lower dental plaque accumulation in the thyroiditis group.

The influence of thyroid hormones on periodontal health has been evaluated in animal studies. Hypothyroidism was documented to enhance periodontitis-related bone loss as a function of an increased number of resorbing cells [7]. Also, clinical studies provided on humans indicated worse periodontal status in patients with thyroid disease. However, only one study describes the impact of thyroid hormones on gingival tissue and hygiene. Venkatesh et al. indicated significantly higher plaque and gingival scores, as well as developmental defects of enamel in children with thyroid disorders, of which children with congenital hypothyroidism constituted 73% [20]. In that study group, children with thyroid disorders had significantly poorer periodontal health due to inadequate oral hygiene compared to healthy control, and only 7% of them had visited a dentist before, compared to 51% of children in the control group. This result is contrary to our observation in which the negative correlation of API with TSH was detected, and T1D patients with thyroiditis had lower plaque accumulation. However, we included adult patients with autoimmune thyroiditis, and no patients with congenital hypothyroidism, as due to the procedure of neonatal screening for hypothyroidism and immediate treatment, most complications of the disease can be prevented, including dental abnormalities. Thyroid hormones play an important role in antioxidant modulation. The most important prooxidants are the reactive oxygen species (ROS) and reactive nitrogen species (RNS) [21,22]. ROS has been shown to be associated with both hyperthyroidism and hypothyroidism. In thyroiditis, low-grade inflammation is induced by impaired nitric oxide availability and increased serum prostaglandins, cytokines, and matrix metalloproteinases (MMPs), ultimately leading to poor periodontal health status and alveolar bone resorption [23].

To our knowledge, there are no studies on the interrelations of gingival health with the comorbidity of two autoimmunologic disorders—T1D and thyroiditis. Microcirculation in T1D may be affected in many tissues, and thus chronic microvascular complications are clinically diagnosed as retinopathy, chronic kidney disease, and neuropathy. Periodontal disease also develops in part due to microvascular alterations caused by chronic hyperglycemia. However, Rogowicz-Frontczak et al. showed a lower frequency of microvascular complications of T1D in patients with thyroid autoimmunity compared to the T1D and no thyroid autoimmunity group [24]. In that study, the TSH level in the group with positive thyroid autoantibodies was significantly higher. On the other hand, Wysocka-Mincewicz et al. showed that the co-existence of T1D and autoimmune thyroiditis in children worsened the status of retinal parameters measured on optical coherence tomography (OCT) and optical coherence tomography angiography (OCTA) [25]. OCT is a non-invasive method of measuring the retinal and choroidal thickness in vivo, enables the assessment of the microvasculature of the retina and choroid and is used in the diagnosis of various retinal vascular diseases, with diabetic retinopathy included. However, as they report worse retinal parameters in children with thyroiditis and T1D and higher TSH levels in this group, it should be noted that this group also presented worse metabolic control of diabetes (higher HbA1c) compared to the control group.

Scardina et al. evaluated the interdental papilla microcirculation in patients with Hashimoto thyroiditis and no other comorbidities that could affect gingival status. They showed a reduced caliber of capillaries, as well as a greater number and tortuosity of capillary loops compared to healthy control [26]. The altered gingival microcirculation may compromise the first line of defense, with increased prostaglandin E, cytokines and oxidative stress leading to periodontitis [27]. Also, Dong et al. observed certain shifts in microbial profile in the saliva of individuals with elevated serum TSH levels, and insulin resistance was speculated to play an important role in the described biochemical and microbial alteration [28]. As the relationship between periodontal and autoimmunologic diseases is bidirectional, it may be stated that chronic systemic inflammation, a key feature of autoimmunological processes, leads to alterations in endothelial function that may clinically present as gingivitis or periodontitis [29].

Again, the TSH level in our study was an independent predictor of dental plaque accumulation, and we showed that gingivitis was less severe in T1D with thyroiditis.

The limitation of our study is the possible impact of the tooth-brushing technique on dental plaque accumulation. Unfortunately, we could not monitor this factor, and therefore this can bias the results.

Our study provides new insight into the incidence of chronic microvascular complications in the comorbidity of two autoimmunologic diseases. More studies are needed to evaluate the underlying pathogenic mechanisms of this finding.

## 5. Conclusions

Autoimmune thyroiditis was associated with lower accumulation of dental plaque and better gingival status in patients with T1D compared to patients with no thyroid disease. TSH, BMI and male sex were independent predictors of dental plaque accumulation in patients with T1D.

Further studies are needed to determine the causality of this finding.

## Figures and Tables

**Table 1:** Characteristics of the study participants. Comparison of subgroup diagnosed with thyroiditis with subgroup without thyroiditis [number or median (interquartile range, IQR)]. Mann-Whitney U test; ^†^ χ^2^ test.

Parameter	All Patients	Subgroup without Thyroiditis, *n* = 196	Subgroup with Thyroiditis, *n* = 68	*p* Value
Age [years]	29 (22–35)	30 (22–35)	26.5 (21.5–32)	0.1
Gender [male/female]	119/145	105/91	14/54	0.00001 ^†^
T1D duration [years]	12 (7–17)	12 (7–17)	12 (7.5–17.5)	0.9
HbA1c [%]	8 (7.3–9.15)	8.1 (7.4–9.4)	7.7 (7.1–9.05)	0.049
Cigarettes [yes/no]	54/210	51/145	3/65	0.0001 ^†^
BMI [kg/m^2^]	23.5 (21.9–26)	24 (21.9–26)	22 (21.8–24.9)	0.02
TSH [mU/l]	1.71 (1.19–2.64)	1.58 (1.14–2.5)	2.15 (1.26–4.62)	0.002
T-Chol [mg/dl]	184 (158–207.5)	185.5 (157–210)	178 (160.5–196.5)	0.6
LDL-Chol [mg/dl]	95.6 (78–117)	96 (76–117)	94.8 (79.8–110.2)	0.8
HDL-Chol [mg/dl]	63 (55–78)	63 (53–79)	63 (58–77)	0.5
TG [mg/dl]	89 (65–120)	87 (66–120)	91 (62.5–109)	0.7
hs-CRP [mg/dl]	1.02 (0.49–2.32)	0.9 (0.5–2.15)	1.5 (0.39–2.92)	0.2
Retinopathy [yes/no]	70/193	54/141	16/52	0.5 ^†^
Neuropathy [yes/no]	23/241	15/181	8/60	0.3 ^†^

**Table 2:** The differences in gingival indices between analyzed subgroups. Median (IQR)]. Mann-Whitney U test.

Parameter	All Patients	Subgroup without Thyroiditis	Subgroup with Thyroiditis	*p* Value
API	0.34 (0.19–0.57)	0.38 (0.21–0.64) Men: 0.44 (0.22–0.65) vs. Women: 0.3 (0.21–0.56); *p* = 0.4	0.26 (0.16–0.5) Men: 0.38 (0.19–0.56) vs. Women: 0.25 (0.16–0.48); *p* = 0.1	0.01
SBI	0.18 (0.04–0.35)	0.18 (0.04–0.35)	0.14 (0.04–0.36)	0.8
GI	1 (0.67–1.04)	1 (0.79–1.04) Men: 1 (0.83–1.06) vs. Women: 1 (0.58–1.04); *p* = 0.8	0.83 (0.42–1) Men: 0.83 (0.42–1) vs. Women: 0.88 (0.5–1.04); *p* = 0.4	0.02

**Table 3:** Correlation between various parameters and API (Spearman’s rank correlation analysis).

	Rs	*p* Value
Age	0.24	0.0001
T1D duration	0.04	0.5
HbA1c	0.18	0.006
BMI	0.22	0.0008
T-Chol	0.17	0.01
LDL-Chol	0.12	0.07
HDL-Chol	−0.04	0.5
TG	0.16	0.02
TSH	−0.2	0.002
hs-CRP	0.17	0.009

**Table 4:** Multivariate linear regression analysis for API. R^2^ = 0.17; *p* = 0.000008.

Variable	Standardized Coefficient β	*p* Value
Gender, male	0.2	0.001
HbA1c	0.13	0.051
BMI	0.19	0.008
TSH	−0.14	0.04
T-Chol	0.08	0.2
hs-CRP	0.1	0.2
Cigarettes	−0.1	0.9

## Data Availability

The datasets generated during and/or analyzed during the current study are available from the corresponding author upon reasonable request.
